# Supplementary material for: Food frequency questionnaire for foods high in sodium: Validation with the triads method
Source: PLoS One. 2023 Jul 3;18(7):e0288123. doi: 10.1371/journal.pone.0288123 (PMC10317220; doi:10.1371/journal.pone.0288123)
Supplement: S1 File — (ZIP) [file pone.0288123.s001.zip › S1_Questionnaire-English_FFQ-FHS.docx]

## **FOOD FREQUENCY QUESTIONNAIRE FOR FOODS HIGH IN SODIUM**

## **(FFQ-FHS)**

| In the last six (6) months, how often have you been consuming the foods listed in this questionnaire? How much is consumed at a time considering the standard portion? | | | |
| --- | --- | --- | --- |
| **FOOD** | **STANDARD PORTION** | **FREQUENCY OF CONSUMPTION** | **QUANTITY CONSUMED AT A TIME** |
| **PROCESSED MEATS** | | | |
| **Bacon** | 1 medium slice | ( ) Rarely/Never ( ) once a month  ( ) 2–3 times a month ( ) once a week  ( ) 2–4 times a week ( ) once daily  ( ) twice daily ( ) ≥3 times a day | ( ) ½ portion  ( ) 1 portion  ( ) 2 portions  ( ) most _____ |
| **Beef jerky** | 1 medium piece | ( ) Rarely/Never ( ) once a month  ( ) 2–3 times a month ( ) once a week  ( ) 2–4 times a week ( ) once daily  ( ) twice daily ( ) ≥3 times a day | ( ) ½ portion  ( ) 1 portion  ( ) 2 portions  ( ) most _____ |
| **Sun dried meat** | 1 medium piece | ( ) Rarely/Never ( ) once a month  ( ) 2–3 times a month ( ) once a week  ( ) 2–4 times a week ( ) once daily  ( ) twice daily ( ) ≥3 times a day | ( ) ½ portion  ( ) 1 portion  ( ) 2 portions  ( ) most _____ |
| **Hamburger meat** | 1 unit | ( ) Rarely/Never ( ) once a month  ( ) 2–3 times a month ( ) once a week  ( ) 2–4 times a week ( ) once daily  ( ) twice daily ( ) ≥3 times a day | ( ) ½ portion  ( ) 1 portion  ( ) 2 portions  ( ) most _____ |
| **Pepperoni/Paio sausage** | 1 medium slice | ( ) Rarely/Never ( ) once a month  ( ) 2–3 times a month ( ) once a week  ( ) 2–4 times a week ( ) once daily  ( ) twice daily ( ) ≥3 times a day | ( ) ½ portion  ( ) 1 portion  ( ) 2 portions  ( ) most _____ |
| **Tuscan sausage** | 1 medium slice | ( ) Rarely/Never ( ) once a month  ( ) 2–3 times a month ( ) once a week  ( ) 2–4 times a week ( ) once daily  ( ) twice daily ( ) ≥3 times a day | ( ) ½ portion  ( ) 1 portion  ( ) 2 portions  ( ) most _____ |
| **Mortadella** | 1 medium slice | ( ) Rarely/Never ( ) once a month  ( ) 2–3 times a month ( ) once a week  ( ) 2–4 times a week ( ) once daily  ( ) twice daily ( ) ≥3 times a day | ( ) ½ portion  ( ) 1 portion  ( ) 2 portions  ( ) most _____ |
| **Frozen seasoned turkey/chicken** | 1 medium slice | ( ) Rarely/Never ( ) once a month  ( ) 2–3 times a month ( ) once a week  ( ) 2–4 times a week ( ) once daily  ( ) twice daily ( ) ≥3 times a day | ( ) ½ portion  ( ) 1 portion  ( ) 2 portions  ( ) most _____ |
| **Ham** | 1 medium slice | ( ) Rarely/Never ( ) once a month  ( ) 2–3 times a month ( ) once a week  ( ) 2–4 times a week ( ) once daily  ( ) twice daily ( ) ≥3 times a day | ( ) ½ portion  ( ) 1 portion  ( ) 2 portions  ( ) most _____ |
| **Sausage** | 1 medium slice | ( ) Rarely/Never ( ) once a month  ( ) 2–3 times a month ( ) once a week  ( ) 2–4 times a week ( ) once daily  ( ) twice daily ( ) ≥3 times a day | ( ) ½ portion  ( ) 1 portion  ( ) 2 portions  ( ) most _____ |

| **FOOD** | **STANDARD PORTION** | **FREQUENCY OF CONSUMPTION** | **QUANTITY CONSUMED AT A TIME** |
| --- | --- | --- | --- |
| **CANNED AND PRESERVED FOODS** | | | |
| **Olive** | 1 unit | ( ) Rarely/Never ( ) once a month  ( ) 2–3 times a month ( ) once a week  ( ) 2–4 times a week ( ) once daily  ( ) twice daily ( ) ≥3 times a day | ( ) ½ portion  ( ) 1 portion  ( ) 2 portions  ( ) most _____ |
| **Pickled mushrooms** | 1 tablespoon | ( ) Rarely/Never ( ) once a month  ( ) 2–3 times a month ( ) once a week  ( ) 2–4 times a week ( ) once daily  ( ) twice daily ( ) ≥3 times a day | ( ) ½ portion  ( ) 1 portion  ( ) 2 s portions  ( ) most _____ |
| **Pickled palm heart** | 1 tablespoon | ( ) Rarely/Never ( ) once a month  ( ) 2–3 times a month ( ) once a week  ( ) 2–4 times a week ( ) once daily  ( ) twice daily ( ) ≥3 times a day | ( ) ½ portion  ( ) 1 portion  ( ) 2 portions  ( ) most _____ |
| **DAIRY PRODUCTS** | | | |
| **Butter with salt** | 1 knife tip | ( ) Rarely/Never ( ) once a month  ( ) 2–3 times a month ( ) once a week  ( ) 2–4 times a week ( ) once daily  ( ) twice daily ( ) ≥3 times a day | ( ) ½ portion  ( ) 1 portion  ( ) 2 portions  ( ) most _____ |
| **Margarine with salt** | 1 knife tip | ( ) Rarely/Never ( ) once a month  ( ) 2–3 times a month ( ) once a week  ( ) 2–4 times a week ( ) once daily  ( ) twice daily ( ) ≥3 times a day | ( ) ½ portion  ( ) 1 portion  ( ) 2 portions  ( ) most _____ |
| **Curd cheese** | 1 medium slice | ( ) Rarely/Never ( ) once a month  ( ) 2–3 times a month ( ) once a week  ( ) 2–4 times a week ( ) once daily  ( ) twice daily ( ) ≥3 times a day | ( ) ½ portion  ( ) 1 portion  ( ) 2 s portions  ( ) most _____ |
| **Butter cheese (curd)** | 1 medium slice | ( ) Rarely/Never ( ) once a month  ( ) 2–3 times a month ( ) once a week  ( ) 2–4 times a week ( ) once daily  ( ) twice daily ( ) ≥3 times a day | ( ) ½ portion  ( ) 1 portion  ( ) 2 portions  ( ) most _____ |
| **Fresh Minas cheese** | 1 medium slice | ( ) Rarely/Never ( ) once a month  ( ) 2–3 times a month ( ) once a week  ( ) 2–4 times a week ( ) once daily  ( ) twice daily ( ) ≥3 times a day | ( ) ½ portion  ( ) 1 portion  ( ) 2 s portions  ( ) most _____ |
| **Mozzarella cheese** | 1 medium slice | ( ) Rarely/Never ( ) once a month  ( ) 2–3 times a month ( ) once a week  ( ) 2–4 times a week ( ) once daily  ( ) twice daily ( ) ≥3 times a day | ( ) ½ portion  ( ) 1 portion  ( ) 2 portions  ( ) most _____ |
| **Cheese plate** | 1 medium slice | ( ) Rarely/Never ( ) once a month  ( ) 2–3 times a month ( ) once a week  ( ) 2–4 times a week ( ) once daily  ( ) twice daily ( ) ≥3 times a day | ( ) ½ portion  ( ) 1 portion  ( ) 2 portions  ( ) most _____ |
| **Grated cheese** | 1 tablespoon | ( ) Rarely/Never ( ) once a month  ( ) 2–3 times a month ( ) once a week  ( ) 2–4 times a week ( ) once daily  ( ) twice daily ( ) ≥3 times a day | ( ) ½ portion  ( ) 1 portion  ( ) 2 portions  ( ) most _____ |
| **Cream cheese** | 1 knife tip | ( ) Rarely/Never ( ) once a month  ( ) 2–3 times a month ( ) once a week  ( ) 2–4 times a week ( ) once daily  ( ) twice daily ( ) ≥3 times a day | ( ) ½ portion  ( ) 1 portion  ( ) 2 portions  ( ) most _____ |

| **FOOD** | **STANDARD PORTION** | **FREQUENCY OF CONSUMPTION** | **QUANTITY CONSUMED AT A TIME** |
| --- | --- | --- | --- |
| **BAKERY AND PASTA** | | | |
| **Cream cracker cookie** | 1 unit | ( ) Rarely/Never ( ) once a month  ( ) 2–3 times a month ( ) once a week  ( ) 2–4 times a week ( ) once daily  ( ) twice daily ( ) ≥3 times a day | ( ) ½ portion  ( ) 1 portion  ( ) 2 portions  ( ) most _____ |
| **Sprinkle biscuit** | 1 thread unit | ( ) Rarely/Never ( ) once a month  ( ) 2–3 times a month ( ) once a week  ( ) 2–4 times a week ( ) once daily  ( ) twice daily ( ) ≥3 times a day | ( ) ½ portion  ( ) 1 portion  ( ) 2 portions  ( ) most _____ |
| **Wholemeal salted cracker** | 1 unit | ( ) Rarely/Never ( ) once a month  ( ) 2–3 times a month ( ) once a week  ( ) 2–4 times a week ( ) once daily  ( ) twice daily ( ) ≥3 times a day | ( ) ½ portion  ( ) 1 portion  ( ) 2 portions  ( ) most _____ |
| **Little tube** | 1 unit | ( ) Rarely/Never ( ) once a month  ( ) 2–3 times a month ( ) once a week  ( ) 2–4 times a week ( ) once daily  ( ) twice daily ( ) ≥3 times a day | ( ) ½ portion  ( ) 1 portion  ( ) 2 portions  ( ) most _____ |
| **Corn breakfast cereal** | 1 small bowl | ( ) Rarely/Never ( ) once a month  ( ) 2–3 times a month ( ) once a week  ( ) 2–4 times a week ( ) once daily  ( ) twice daily ( ) ≥3 times a day | ( ) ½ portion  ( ) 1 portion  ( ) 2 portions  ( ) most _____ |
| **Hot dog bun** | 1 unit | ( ) Rarely/Never ( ) once a month  ( ) 2–3 times a month ( ) once a week  ( ) 2–4 times a week ( ) once daily  ( ) twice daily ( ) ≥3 times a day | ( ) ½ portion  ( ) 1 portion  ( ) 2 portions  ( ) most _____ |
| **Loaf bread** | 1 slice | ( ) Rarely/Never ( ) once a month  ( ) 2–3 times a month ( ) once a week  ( ) 2–4 times a week ( ) once daily  ( ) twice daily ( ) ≥3 times a day | ( ) ½ portion  ( ) 1 portion  ( ) 2 portions  ( ) most _____ |
| **Wholemeal bread** | 1 slice | ( ) Rarely/Never ( ) once a month  ( ) 2–3 times a month ( ) once a week  ( ) 2–4 times a week ( ) once daily  ( ) twice daily ( ) ≥3 times a day | ( ) ½ portion  ( ) 1 portion  ( ) 2 portions  ( ) most _____ |
| **Hamburger bread** | 1 unit | ( ) Rarely/Never ( ) once a month  ( ) 2–3 times a month ( ) once a week  ( ) 2–4 times a week ( ) once daily  ( ) twice daily ( ) ≥3 times a day | ( ) ½ portion  ( ) 1 portion  ( ) 2 portions  ( ) most _____ |
| **Milk bread** | 1 slice | ( ) Rarely/Never ( ) once a month  ( ) 2–3 times a month ( ) once a week  ( ) 2–4 times a week ( ) once daily  ( ) twice daily ( ) ≥3 times a day | ( ) ½ portion  ( ) 1 portion  ( ) 2 portions  ( ) most _____ |
| **French bread** | 1 unit | ( ) Rarely/Never ( ) once a month  ( ) 2–3 times a month ( ) once a week  ( ) 2–4 times a week ( ) once daily  ( ) twice daily ( ) ≥3 times a day | ( ) ½ portion  ( ) 1 portion  ( ) 2 portions  ( ) most _____ |
| **Tortilla bread** | 1 unit | ( ) Rarely/Never ( ) once a month  ( ) 2–3 times a month ( ) once a week  ( ) 2–4 times a week ( ) once daily  ( ) twice daily ( ) ≥3 times a day | ( ) ½ portion  ( ) 1 portion  ( ) 2 portions  ( ) most _____ |
| **French bread toast** | 1 unit | ( ) Rarely/Never ( ) once a month  ( ) 2–3 times a month ( ) once a week  ( ) 2–4 times a week ( ) once daily  ( ) twice daily ( ) ≥3 times a day | ( ) ½ portion  ( ) 1 portion  ( ) 2 portions  ( ) most _____ |

| **FOOD**  **(continuation)** | **STANDARD PORTION** | **FREQUENCY OF CONSUMPTION** | **QUANTITY CONSUMED AT A TIME** |
| --- | --- | --- | --- |
| **Industrialized toast** | 1 unit | ( ) Rarely/Never ( ) once a month  ( ) 2–3 times a month ( ) once a week  ( ) 2–4 times a week ( ) once daily  ( ) twice daily ( ) ≥3 times a day | ( ) ½ portion  ( ) 1 portion  ( ) 2 portions  ( ) most _____ |
| **SALTS AND CONDIMENTS** | | | |
| **Ketchup** | 1 tablespoon | ( ) Rarely/Never ( ) once a month  ( ) 2–3 times a month ( ) once a week  ( ) 2–4 times a week ( ) once daily  ( ) twice daily ( ) ≥3 times a day | ( ) ½ portion  ( ) 1 portion  ( ) 2 portions  ( ) most _____ |
| **Mayonnaise** | 1 knife tip | ( ) Rarely/Never ( ) once a month  ( ) 2–3 times a month ( ) once a week  ( ) 2–4 times a week ( ) once daily  ( ) twice daily ( ) ≥3 times a day | ( ) ½ portion  ( ) 1 portion  ( ) 2 portions  ( ) most _____ |
| **Industrialized tomato sauce** | 1 tablespoon | ( ) Rarely/Never ( ) once a month  ( ) 2–3 times a month ( ) once a week  ( ) 2–4 times a week ( ) once daily  ( ) twice daily ( ) ≥3 times a day | ( ) ½ portion  ( ) 1 portion  ( ) 2 portions  ( ) most _____ |
| **MISCELLANEOUS** | | | |
| **Potato sticks** | 1/2 cup | ( ) Rarely/Never ( ) once a month  ( ) 2–3 times a month ( ) once a week  ( ) 2–4 times a week ( ) once daily  ( ) twice daily ( ) ≥3 times a day | ( ) ½ portion  ( ) 1 portion  ( ) 2 portions  ( ) most _____ |
| **Bauru**  **(roasted salty)** | 1 medium unit | ( ) Rarely/Never ( ) once a month  ( ) 2–3 times a month ( ) once a week  ( ) 2–4 times a week ( ) once daily  ( ) twice daily ( ) ≥3 times a day | ( ) ½ portion  ( ) 1 portion  ( ) 2 portions  ( ) most _____ |
| ***Capeletti* with chicken stuffing** | 1 shallow dish | ( ) Rarely/Never ( ) once a month  ( ) 2–3 times a month ( ) once a week  ( ) 2–4 times a week ( ) once daily  ( ) twice daily ( ) ≥3 times a day | ( ) ½ portion  ( ) 1 portion  ( ) 2 portions  ( ) most _____ |
| **Chicken drumstick** | 1 medium unit | ( ) Rarely/Never ( ) once a month  ( ) 2–3 times a month ( ) once a week  ( ) 2–4 times a week ( ) once daily  ( ) twice daily ( ) ≥3 times a day | ( ) ½ portion  ( ) 1 portion  ( ) 2 portions  ( ) most _____ |
| **Oven pastel** | 1 unit | ( ) Rarely/Never ( ) once a month  ( ) 2–3 times a month ( ) once a week  ( ) 2–4 times a week ( ) once daily  ( ) twice daily ( ) ≥3 times a day | ( ) ½ portion  ( ) 1 portion  ( ) 2 portions  ( ) most _____ |
| **Peanut butter** | 1 knife tip | ( ) Rarely/Never ( ) once a month  ( ) 2–3 times a month ( ) once a week  ( ) 2–4 times a week ( ) once daily  ( ) twice daily ( ) ≥3 times a day | ( ) ½ portion  ( ) 1 portion  ( ) 2 portions  ( ) most _____ |
| **Meat roulade** | 1 medium slice | ( ) Rarely/Never ( ) once a month  ( ) 2–3 times a month ( ) once a week  ( ) 2–4 times a week ( ) once daily  ( ) twice daily ( ) ≥3 times a day | ( ) ½ portion  ( ) 1 portion  ( ) 2 portions  ( ) most _____ |
| **Corn snack** | 1 medium pack | ( ) Rarely/Never ( ) once a month  ( ) 2–3 times a month ( ) once a week  ( ) 2–4 times a week ( ) once daily  ( ) twice daily ( ) ≥3 times a day | ( ) ½ portion  ( ) 1 portion  ( ) 2 portions  ( ) most _____ |

| **FOOD** | **STANDARD PORTION** | **FREQUENCY OF CONSUMPTION** | **QUANTITY CONSUMED AT A TIME** |
| --- | --- | --- | --- |
| **REGIONAL FOODS** | | | |
| **Cooked rice** | 1 spoon of rice | ( ) Rarely/Never ( ) once a month  ( ) 2–3 times a month ( ) once a week  ( ) 2–4 times a week ( ) once daily  ( ) twice daily ( ) ≥3 times a day | ( ) ½ portion  ( ) 1 portion  ( ) 2 portions  ( ) most _____ |
| **Boiled sweet potato** | 1 medium slice | ( ) Rarely/Never ( ) once a month  ( ) 2–3 times a month ( ) once a week  ( ) 2–4 times a week ( ) once daily  ( ) twice daily ( ) ≥3 times a day | ( ) ½ portion  ( ) 1 portion  ( ) 2 portions  ( ) most _____ |
| **Beef** | 1 medium steak | ( ) Rarely/Never ( ) once a month  ( ) 2–3 times a month ( ) once a week  ( ) 2–4 times a week ( ) once daily  ( ) twice daily ( ) ≥3 times a day | ( ) ½ portion  ( ) 1 portion  ( ) 2 portions  ( ) most _____ |
| **Baked beans** | 1 medium shell | ( ) Rarely/Never ( ) once a month  ( ) 2–3 times a month ( ) once a week  ( ) 2–4 times a week ( ) once daily  ( ) twice daily ( ) ≥3 times a day | ( ) ½ portion  ( ) 1 portion  ( ) 2 portions  ( ) most _____ |
| **Chicken** | 1 medium fillet | ( ) Rarely/Never ( ) once a month  ( ) 2–3 times a month ( ) once a week  ( ) 2–4 times a week ( ) once daily  ( ) twice daily ( ) ≥3 times a day | ( ) ½ portion  ( ) 1 portion  ( ) 2 portions  ( ) most _____ |
| **Yam** | 1 medium piece | ( ) Rarely/Never ( ) once a month  ( ) 2–3 times a month ( ) once a week  ( ) 2–4 times a week ( ) once daily  ( ) twice daily ( ) ≥3 times a day | ( ) ½ portion  ( ) 1 portion  ( ) 2 portions  ( ) most _____ |
| **Boiled cassava** | 1 medium piece | ( ) Rarely/Never ( ) once a month  ( ) 2–3 times a month ( ) once a week  ( ) 2–4 times a week ( ) once daily  ( ) twice daily ( ) ≥3 times a day | ( ) ½ portion  ( ) 1 portion  ( ) 2 portions  ( ) most _____ |
| **Fish** | 1 medium fillet | ( ) Rarely/Never ( ) once a month  ( ) 2–3 times a month ( ) once a week  ( ) 2–4 times a week ( ) once daily  ( ) twice daily ( ) ≥3 times a day | ( ) ½ portion  ( ) 1 portion  ( ) 2 portions  ( ) most _____ |
