# Supplementary material for: Food frequency questionnaire for foods high in sodium: Validation with the triads method
Source: PLoS One. 2023 Jul 3;18(7):e0288123. doi: 10.1371/journal.pone.0288123 (PMC10317220; doi:10.1371/journal.pone.0288123)
Supplement: S1 File — (ZIP) [file pone.0288123.s001.zip › S2_Questionario-Portugues_QFA-ATS.docx]

## **QUESTIONÁRIO DE FREQUÊNCIA ALIMENTAR DE ALIMENTOS COM ALTO TEOR DE SÓDIO (QFA-ATS)**

| Nos últimos seis (6) meses com que frequência você está consumindo os alimentos listados nesse questionário? Qual a quantidade consumida por vez considerando a porção padrão? | | | |
| --- | --- | --- | --- |
| **ALIMENTO** | **PORÇÃO PADRÃO** | **FREQUÊNCIA DE CONSUMO** | **QUANTIDADE CONSUMIDA POR VEZ** |
| **CARNES INDUSTRIALIZADAS** | | | |
| **Bacon** | 1 fatia média | ( ) Raramente/Nunca ( ) 1x/mês  ( ) 2 a 3x/mês ( ) 1x/sem ( ) 2 a 4x/sem  ( ) 1x/dia ( ) 2x/dia ( ) ≥ 3x/dia | ( ) ½ porção  ( ) 1 porção  ( ) 2 porções  ( ) mais _____ |
| **Carne de charque** | 1 pedaço médio | ( ) Raramente/Nunca ( ) 1x/mês  ( ) 2 a 3x/mês ( ) 1x/sem ( ) 2 a 4x/sem  ( ) 1x/dia ( ) 2x/dia ( ) ≥ 3x/dia | ( ) ½ porção  ( ) 1 porção  ( ) 2 porções  ( ) mais _____ |
| **Carne de sol** | 1 pedaço médio | ( ) Raramente/Nunca ( ) 1x/mês  ( ) 2 a 3x/mês ( ) 1x/sem ( ) 2 a 4x/sem  ( ) 1x/dia ( ) 2x/dia ( ) ≥ 3x/dia | ( ) ½ porção  ( ) 1 porção  ( )2 porções  ( ) mais _____ |
| **Carne de hambúrguer** | 1 unidade | ( ) Raramente/Nunca ( ) 1x/mês  ( ) 2 a 3x/mês ( ) 1x/sem ( ) 2 a 4x/sem  ( ) 1x/dia ( ) 2x/dia ( ) ≥ 3x/dia | ( ) ½ porção  ( ) 1 porção  ( ) 2 porções  ( ) mais _____ |
| **Linguiça calabresa/Paio** | 1 fatia média | ( ) Raramente/Nunca ( ) 1x/mês  ( ) 2 a 3x/mês ( ) 1x/sem ( ) 2 a 4x/sem  ( ) 1x/dia ( ) 2x/dia ( ) ≥ 3x/dia | ( ) ½ porção  ( ) 1 porção  ( ) 2 porções  ( ) mais _____ |
| **Linguiça toscana** | 1 fatia média | ( ) Raramente/Nunca ( ) 1x/mês  ( ) 2 a 3x/mês ( ) 1x/sem ( ) 2 a 4x/sem  ( ) 1x/dia ( ) 2x/dia ( ) ≥ 3x/dia | ( ) ½ porção  ( ) 1 porção  ( ) 2 porções  ( ) mais _____ |
| **Mortadela** | 1 fatia média | ( ) Raramente/Nunca ( ) 1x/mês  ( ) 2 a 3x/mês ( ) 1x/sem ( ) 2 a 4x/sem  ( ) 1x/dia ( ) 2x/dia ( ) ≥ 3x/dia | ( ) ½ porção  ( ) 1 porção  ( ) 2 porções  ( ) mais _____ |
| **Peru/Frango temperado congelado** | 1 fatia média | ( ) Raramente/Nunca ( ) 1x/mês  ( ) 2 a 3x/mês ( ) 1x/sem ( ) 2 a 4x/sem  ( ) 1x/dia ( ) 2x/dia ( ) ≥ 3x/dia | ( ) ½ porção  ( ) 1 porção  ( ) 2 porções  ( ) mais _____ |
| **Presunto** | 1 fatia média | ( ) Raramente/Nunca ( ) 1x/mês  ( ) 2 a 3x/mês ( ) 1x/sem ( ) 2 a 4x/sem  ( ) 1x/dia ( ) 2x/dia ( ) ≥ 3x/dia | ( ) ½ porção  ( ) 1 porção  ( ) 2 porções  ( ) mais _____ |
| **Salsicha** | 1 fatia média | ( ) Raramente/Nunca ( ) 1x/mês  ( ) 2 a 3x/mês ( ) 1x/sem ( ) 2 a 4x/sem  ( ) 1x/dia ( ) 2x/dia ( ) ≥ 3x/dia | ( ) ½ porção  ( ) 1 porção  ( ) 2 porções  ( ) mais _____ |

| **ALIMENTO** | **PORÇÃO PADRÃO** | **FREQUÊNCIA DE CONSUMO** | **QUANTIDADE CONSUMIDA POR VEZ** |
| --- | --- | --- | --- |
| **ENLATADOS E CONSERVAS** | | | |
| **Azeitona** | 1 unidade | ( ) Raramente/Nunca ( ) 1x/mês  ( ) 2 a 3x/mês ( ) 1x/sem ( ) 2 a 4x/sem  ( ) 1x/dia ( ) 2x/dia ( ) ≥ 3x/dia | ( ) ½ porção  ( ) 1 porção  ( ) 2 porções  ( ) mais _____ |
| **Champignon em conserva** | 1 colher de sopa | ( ) Raramente/Nunca ( ) 1x/mês  ( ) 2 a 3x/mês ( ) 1x/sem ( ) 2 a 4x/sem  ( ) 1x/dia ( ) 2x/dia ( ) ≥ 3x/dia | ( ) ½ porção  ( ) 1 porção  ( ) 2 porções  ( ) mais _____ |
| **Palmito em conserva** | 1 colher de sopa | ( ) Raramente/Nunca ( ) 1x/mês  ( ) 2 a 3x/mês ( ) 1x/sem ( ) 2 a 4x/sem  ( ) 1x/dia ( ) 2x/dia ( ) ≥ 3x/dia | ( ) ½ porção  ( ) 1 porção  ( ) 2 porções  ( ) mais _____ |
| **LATICÍNIOS** | | | |
| **Manteiga com sal** | 1 ponta de faca | ( ) Raramente/Nunca ( ) 1x/mês  ( ) 2 a 3x/mês ( ) 1x/sem ( ) 2 a 4x/sem  ( ) 1x/dia ( ) 2x/dia ( ) ≥ 3x/dia | ( ) ½ porção  ( ) 1 porção  ( ) 2 porções  ( ) mais _____ |
| **Margarina com sal** | 1 ponta de faca | ( ) Raramente/Nunca ( ) 1x/mês  ( ) 2 a 3x/mês ( ) 1x/sem ( ) 2 a 4x/sem  ( ) 1x/dia ( ) 2x/dia ( ) ≥ 3x/dia | ( ) ½ porção  ( ) 1 porção  ( ) 2 porções  ( ) mais _____ |
| **Queijo de coalho** | 1 fatia média | ( ) Raramente/Nunca ( ) 1x/mês  ( ) 2 a 3x/mês ( ) 1x/sem ( ) 2 a 4x/sem  ( ) 1x/dia ( ) 2x/dia ( ) ≥ 3x/dia | ( ) ½ porção  ( ) 1 porção  ( ) 2 porções  ( ) mais _____ |
| **Queijo de manteiga (requeijão)** | 1 fatia média | ( ) Raramente/Nunca ( ) 1x/mês  ( ) 2 a 3x/mês ( ) 1x/sem ( ) 2 a 4x/sem  ( ) 1x/dia ( ) 2x/dia ( ) ≥ 3x/dia | ( ) ½ porção  ( ) 1 porção  ( ) 2 porções  ( ) mais _____ |
| **Queijo minas frescal** | 1 fatia média | ( ) Raramente/Nunca ( ) 1x/mês  ( ) 2 a 3x/mês ( ) 1x/sem ( ) 2 a 4x/sem  ( ) 1x/dia ( ) 2x/dia ( ) ≥ 3x/dia | ( ) ½ porção  ( ) 1 porção  ( ) 2 porções  ( ) mais _____ |
| **Queijo muçarela** | 1 fatia média | ( ) Raramente/Nunca ( ) 1x/mês  ( ) 2 a 3x/mês ( ) 1x/sem ( ) 2 a 4x/sem  ( ) 1x/dia ( ) 2x/dia ( ) ≥ 3x/dia | ( ) ½ porção  ( ) 1 porção  ( )2 porções  ( ) mais _____ |
| **Queijo prato** | 1 fatia média | ( ) Raramente/Nunca ( ) 1x/mês  ( ) 2 a 3x/mês ( ) 1x/sem ( ) 2 a 4x/sem  ( ) 1x/dia ( ) 2x/dia ( ) ≥ 3x/dia | ( ) ½ porção  ( ) 1 porção  ( ) 2 porções  ( ) mais _____ |
| **Queijo ralado** | 1 colher de sopa | ( ) Raramente/Nunca ( ) 1x/mês  ( ) 2 a 3x/mês ( ) 1x/sem ( ) 2 a 4x/sem  ( ) 1x/dia ( ) 2x/dia ( ) ≥ 3x/dia | ( ) ½ porção  ( ) 1 porção  ( ) 2 porções  ( ) mais _____ |
| **Requeijão cremoso** | 1 ponta de faca | ( ) Raramente/Nunca ( ) 1x/mês  ( ) 2 a 3x/mês ( ) 1x/sem ( ) 2 a 4x/sem  ( ) 1x/dia ( ) 2x/dia ( ) ≥ 3x/dia | ( ) ½ porção  ( ) 1 porção  ( ) 2 porções  ( ) mais _____ |

| **ALIMENTO** | **PORÇÃO PADRÃO** | **FREQUÊNCIA DE CONSUMO** | **QUANTIDADE CONSUMIDA POR VEZ** |
| --- | --- | --- | --- |
| **PANIFICAÇÃO E MASSAS** | | | |
| **Biscoito *cream cracker*** | 1 unidade | ( ) Raramente/Nunca ( ) 1x/mês  ( ) 2 a 3x/mês ( ) 1x/sem ( ) 2 a 4x/sem  ( ) 1x/dia ( ) 2x/dia ( ) ≥ 3x/dia | ( ) ½ porção  ( ) 1 porção  ( ) 2 porções  ( ) mais _____ |
| **Biscoito de polvilho** | 1 unidade rosca | ( ) Raramente/Nunca ( ) 1x/mês  ( ) 2 a 3x/mês ( ) 1x/sem ( ) 2 a 4x/sem  ( ) 1x/dia ( ) 2x/dia ( ) ≥ 3x/dia | ( ) ½ porção  ( ) 1 porção  ( ) 2 porções  ( ) mais _____ |
| **Biscoito salgado integral** | 1 unidade | ( ) Raramente/Nunca ( ) 1x/mês  ( ) 2 a 3x/mês ( ) 1x/sem ( ) 2 a 4x/sem  ( ) 1x/dia ( ) 2x/dia ( ) ≥ 3x/dia | ( ) ½ porção  ( ) 1 porção  ( ) 2 porções  ( ) mais _____ |
| **Bisnaguinha** | 1 unidade | ( ) Raramente/Nunca ( ) 1x/mês  ( ) 2 a 3x/mês ( ) 1x/sem ( ) 2 a 4x/sem  ( ) 1x/dia ( ) 2x/dia ( ) ≥ 3x/dia | ( ) ½ porção  ( ) 1 porção  ( ) 2 porções  ( ) mais _____ |
| **Cereal matinal de milho** | 1 tigela pequena | ( ) Raramente/Nunca ( ) 1x/mês  ( ) 2 a 3x/mês ( ) 1x/sem ( ) 2 a 4x/sem  ( ) 1x/dia ( ) 2x/dia ( ) ≥ 3x/dia | ( ) ½ porção  ( ) 1 porção  ( ) 2 porções  ( ) mais _____ |
| **Pão de cachorro quente** | 1 unidade | ( ) Raramente/Nunca ( ) 1x/mês  ( ) 2 a 3x/mês ( ) 1x/sem ( ) 2 a 4x/sem  ( ) 1x/dia ( ) 2x/dia ( ) ≥ 3x/dia | ( ) ½ porção  ( ) 1 porção  ( ) 2 porções  ( ) mais _____ |
| **Pão de forma** | 1 fatia | ( ) Raramente/Nunca ( ) 1x/mês  ( ) 2 a 3x/mês ( ) 1x/sem ( ) 2 a 4x/sem  ( ) 1x/dia ( ) 2x/dia ( ) ≥ 3x/dia | ( ) ½ porção  ( ) 1 porção  ( ) 2 porções  ( ) mais _____ |
| **Pão de forma integral** | 1 fatia | ( ) Raramente/Nunca ( ) 1x/mês  ( ) 2 a 3x/mês ( ) 1x/sem ( ) 2 a 4x/sem  ( ) 1x/dia ( ) 2x/dia ( ) ≥ 3x/dia | ( ) ½ porção  ( ) 1 porção  ( ) 2 porções  ( ) mais _____ |
| **Pão de hambúrguer** | 1 unidade | ( ) Raramente/Nunca ( ) 1x/mês  ( ) 2 a 3x/mês ( ) 1x/sem ( ) 2 a 4x/sem  ( ) 1x/dia ( ) 2x/dia ( ) ≥ 3x/dia | ( ) ½ porção  ( ) 1 porção  ( ) 2 porções  ( ) mais _____ |
| **Pão de leite** | 1 fatia | ( ) Raramente/Nunca ( ) 1x/mês  ( ) 2 a 3x/mês ( ) 1x/sem ( ) 2 a 4x/sem  ( ) 1x/dia ( ) 2x/dia ( ) ≥ 3x/dia | ( ) ½ porção  ( ) 1 porção  ( ) 2 porções  ( ) mais _____ |
| **Pão francês** | 1 unidade | ( ) Raramente/Nunca ( ) 1x/mês  ( ) 2 a 3x/mês ( ) 1x/sem ( ) 2 a 4x/sem  ( ) 1x/dia ( ) 2x/dia ( ) ≥ 3x/dia | ( ) ½ porção  ( ) 1 porção  ( ) 2 porções  ( ) mais _____ |
| **Pão tipo tortilha** | 1 unidade | ( ) Raramente/Nunca ( ) 1x/mês  ( ) 2 a 3x/mês ( ) 1x/sem ( ) 2 a 4x/sem  ( ) 1x/dia ( ) 2x/dia ( ) ≥ 3x/dia | ( ) ½ porção  ( ) 1 porção  ( ) 2 porções  ( ) mais _____ |
| **Torrada de pão francês** | 1 unidade | ( ) Raramente/Nunca ( ) 1x/mês  ( ) 2 a 3x/mês ( ) 1x/sem ( ) 2 a 4x/sem  ( ) 1x/dia ( ) 2x/dia ( ) ≥ 3x/dia | ( ) ½ porção  ( ) 1 porção  ( ) 2 porções  ( ) mais _____ |

| **ALIMENTO**  **(*continuação*)** | **PORÇÃO PADRÃO** | **FREQUÊNCIA DE CONSUMO** | **QUANTIDADE CONSUMIDA POR VEZ** |
| --- | --- | --- | --- |
| **Torrada industrializada** | 1 unidade | ( ) Raramente/Nunca ( ) 1x/mês  ( ) 2 a 3x/mês ( ) 1x/sem ( ) 2 a 4x/sem  ( ) 1x/dia ( ) 2x/dia ( ) ≥ 3x/dia | ( ) ½ porção  ( ) 1 porção  ( ) 2 porções  ( ) mais _____ |
| **SAIS E CONDIMENTOS** | | | |
| **Catchup** | 1 colher de sopa | ( ) Raramente/Nunca ( ) 1x/mês  ( ) 2 a 3x/mês ( ) 1x/sem ( ) 2 a 4x/sem  ( ) 1x/dia ( ) 2x/dia ( ) ≥ 3x/dia | ( ) ½ porção  ( ) 1 porção  ( ) 2 porções  ( ) mais _____ |
| **Maionese** | 1 ponta de faca | ( ) Raramente/Nunca ( ) 1x/mês  ( ) 2 a 3x/mês ( ) 1x/sem ( ) 2 a 4x/sem  ( ) 1x/dia ( ) 2x/dia ( ) ≥ 3x/dia | ( ) ½ porção  ( ) 1 porção  ( ) 2 porções  ( ) mais _____ |
| **Molho de tomate industrializado** | 1 colher de sopa | ( ) Raramente/Nunca ( ) 1x/mês  ( ) 2 a 3x/mês ( ) 1x/sem ( ) 2 a 4x/sem  ( ) 1x/dia ( ) 2x/dia ( ) ≥ 3x/dia | ( ) ½ porção  ( ) 1 porção  ( ) 2 porções  ( ) mais _____ |
| **MISCELÂNEA** | | | |
| **Batata palha** | 1/2 xícara | ( ) Raramente/Nunca ( ) 1x/mês  ( ) 2 a 3x/mês ( ) 1x/sem ( ) 2 a 4x/sem  ( ) 1x/dia ( ) 2x/dia ( ) ≥ 3x/dia | ( ) ½ porção  ( ) 1 porção  ( ) 2 porções  ( ) mais _____ |
| **Bauru**  **(salgado assado)** | 1 unidade média | ( ) Raramente/Nunca ( ) 1x/mês  ( ) 2 a 3x/mês ( ) 1x/sem ( ) 2 a 4x/sem  ( ) 1x/dia ( ) 2x/dia ( ) ≥ 3x/dia | ( ) ½ porção  ( ) 1 porção  ( ) 2 porções  ( ) mais _____ |
| ***Capeletti* com recheio de frango** | 1 prato raso | ( ) Raramente/Nunca ( ) 1x/mês  ( ) 2 a 3x/mês ( ) 1x/sem ( ) 2 a 4x/sem  ( ) 1x/dia ( ) 2x/dia ( ) ≥ 3x/dia | ( ) ½ porção  ( ) 1 porção  ( ) 2 porções  ( ) mais _____ |
| **Coxinha de frango** | 1 unidade média | ( ) Raramente/Nunca ( ) 1x/mês  ( ) 2 a 3x/mês ( ) 1x/sem ( ) 2 a 4x/sem  ( ) 1x/dia ( ) 2x/dia ( ) ≥ 3x/dia | ( ) ½ porção  ( ) 1 porção  ( ) 2 porções  ( ) mais _____ |
| **Pastel de forno** | 1 unidade | ( ) Raramente/Nunca ( ) 1x/mês  ( ) 2 a 3x/mês ( ) 1x/sem ( ) 2 a 4x/sem  ( ) 1x/dia ( ) 2x/dia ( ) ≥ 3x/dia | ( ) ½ porção  ( ) 1 porção  ( ) 2 porções  ( ) mais _____ |
| **Pasta de amendoim** | 1 ponta de faca | ( ) Raramente/Nunca ( ) 1x/mês  ( ) 2 a 3x/mês ( ) 1x/sem ( ) 2 a 4x/sem  ( ) 1x/dia ( ) 2x/dia ( ) ≥ 3x/dia | ( ) ½ porção  ( ) 1 porção  ( ) 2 porções  ( ) mais _____ |
| **Rocambole de carne** | 1 fatia média | ( ) Raramente/Nunca ( ) 1x/mês  ( ) 2 a 3x/mês ( ) 1x/sem ( ) 2 a 4x/sem  ( ) 1x/dia ( ) 2x/dia ( ) ≥ 3x/dia | ( ) ½ porção  ( ) 1 porção  ( ) 2 porções  ( ) mais _____ |
| **Salgadinho de milho** | 1 pacote médio | ( ) Raramente/Nunca ( ) 1x/mês  ( ) 2 a 3x/mês ( ) 1x/sem ( ) 2 a 4x/sem  ( ) 1x/dia ( ) 2x/dia ( ) ≥ 3x/dia | ( ) ½ porção  ( ) 1 porção  ( ) 2 porções  ( ) mais _____ |

| **ALIMENTO** | **PORÇÃO PADRÃO** | **FREQUÊNCIA DE CONSUMO** | **QUANTIDADE CONSUMIDA POR VEZ** |
| --- | --- | --- | --- |
| **REGIONAIS** | | | |
| **Arroz cozido** | 1 colher de arroz | ( ) Raramente/Nunca ( ) 1x/mês  ( ) 2 a 3x/mês ( ) 1x/sem ( ) 2 a 4x/sem  ( ) 1x/dia ( ) 2x/dia ( ) ≥ 3x/dia | ( ) ½ porção  ( ) 1 porção  ( ) 2 porções  ( ) mais _____ |
| **Batata-doce cozida** | 1 fatia média | ( ) Raramente/Nunca ( ) 1x/mês  ( ) 2 a 3x/mês ( ) 1x/sem ( ) 2 a 4x/sem  ( ) 1x/dia ( ) 2x/dia ( ) ≥ 3x/dia | ( ) ½ porção  ( ) 1 porção  ( ) 2 porções  ( ) mais _____ |
| **Carne bovina** | 1 bife médio | ( ) Raramente/Nunca ( ) 1x/mês  ( ) 2 a 3x/mês ( ) 1x/sem ( ) 2 a 4x/sem  ( ) 1x/dia ( ) 2x/dia ( ) ≥ 3x/dia | ( ) ½ porção  ( ) 1 porção  ( ) 2 porções  ( ) mais _____ |
| **Feijão cozido** | 1 concha média | ( ) Raramente/Nunca ( ) 1x/mês  ( ) 2 a 3x/mês ( ) 1x/sem ( ) 2 a 4x/sem  ( ) 1x/dia ( ) 2x/dia ( ) ≥ 3x/dia | ( ) ½ porção  ( ) 1 porção  ( ) 2 porções  ( ) mais _____ |
| **Frango** | 1 filé médio | ( ) Raramente/Nunca ( ) 1x/mês  ( ) 2 a 3x/mês ( ) 1x/sem ( ) 2 a 4x/sem  ( ) 1x/dia ( ) 2x/dia ( ) ≥ 3x/dia | ( ) ½ porção  ( ) 1 porção  ( ) 2 porções  ( ) mais _____ |
| **Inhame** | 1 pedaço médio | ( ) Raramente/Nunca ( ) 1x/mês  ( ) 2 a 3x/mês ( ) 1x/sem ( ) 2 a 4x/sem  ( ) 1x/dia ( ) 2x/dia ( ) ≥ 3x/dia | ( ) ½ porção  ( ) 1 porção  ( ) 2 porções  ( ) mais _____ |
| **Macaxeira cozida** | 1 pedaço médio | ( ) Raramente/Nunca ( ) 1x/mês  ( ) 2 a 3x/mês ( ) 1x/sem ( ) 2 a 4x/sem  ( ) 1x/dia ( ) 2x/dia ( ) ≥ 3x/dia | ( ) ½ porção  ( ) 1 porção  ( ) 2 porções  ( ) mais _____ |
| **Peixe** | 1 filé médio | ( ) Raramente/Nunca ( ) 1x/mês  ( ) 2 a 3x/mês ( ) 1x/sem ( ) 2 a 4x/sem  ( ) 1x/dia ( ) 2x/dia ( ) ≥ 3x/dia | ( ) ½ porção  ( ) 1 porção  ( ) 2 porções  ( ) mais _____ |
