# Supplementary material for: Food frequency questionnaire for foods high in sodium: Validation with the triads method
Source: PLoS One. 2023 Jul 3;18(7):e0288123. doi: 10.1371/journal.pone.0288123 (PMC10317220; doi:10.1371/journal.pone.0288123)
Supplement: Attachment — Submitted filename: Response to Reviewers.docx [file pone.0288123.s002.docx]

**Response to Reviewers**

Journal Requirements:

1. Please ensure that your manuscript meets PLOS ONE's style requirements, including those for file naming. The PLOS ONE style templates can be found at

<https://journals.plos.org/plosone/s/file?id=wjVg/PLOSOne_formatting_sample_main_body.pdf> and

<https://journals.plos.org/plosone/s/file?id=ba62/PLOSOne_formatting_sample_title_authors_affiliations.pdf>

**Response:** The suggested changes have been made.

2. We suggest you thoroughly copyedit your manuscript for language usage, spelling, and grammar. If you do not know anyone who can help you do this, you may wish to consider employing a professional scientific editing service.

**Response:** The manuscript has been edited by Editage to ensure language and grammatical accuracy.

3. Please include additional information regarding the survey or questionnaire used in the study and ensure that you have provided sufficient details that others could replicate the analyses. For instance, if you developed a questionnaire as part of this study and it is not under a copyright more restrictive than CC-BY, please include a copy, in both the original language and English, as Supporting Information.

**Response:** As requested, a copy in the original language and English of the constructed FFQ has been attached.

4. Please provide additional details regarding participant consent. In the Methods section, please ensure that you have specified (1) whether consent was informed and (2) what type you obtained (for instance, written or verbal). If your study included minors, state whether you obtained consent from parents or guardians. If the need for consent was waived by the ethics committee, please include this information.

**Response:** The suggested changes have been made.

5. We note that the grant information you provided in the ‘Funding Information’ and ‘Financial Disclosure’ sections do not match.

When you resubmit, please ensure that you provide the correct grant numbers for the awards you received for your study in the ‘Funding Information’ section.

**Response:** This study was partially financed by the following:

1. Scholarship awarded by the Fundação de Apoio à Pesquisa e a Inovação Tecnológica do Estado de Sergipe (Fapitec/SE/Brasil) [Research and Technological Innovation Support Foundation in the State of Sergipe]

<https://fapitec.se.gov.br/>

2. Financial resources granted by the Coordenação de Aperfeiçoamento de Pessoal de Nível Superior - Brasil (CAPES/Brasil) [Coordination for the Improvement of Higher Education Personnel], financing code 001, PROMOB, process no. 88881.157882/2017-01

<https://www.gov.br/capes/pt-br>

The funders had no role in the study design, data collection and analysis, decision to publish, or manuscript preparation. No additional external funding was received for the study.

6. Please provide an amended statement that declares *all* the funding or sources of support (whether external or internal to your organization) received during this study, as detailed online in our guide for authors at http://journals.plos.org/plosone/s/submit-now. Please also include the statement “There was no additional external funding received for this study.” in your updated Funding Statement.

Please include your amended Funding Statement within your cover letter. We will change the online submission form on your behalf.

**Response:** The suggested changes have been made.

7. We note that you have provided funding information that is not currently declared in your Funding Statement. However, funding information should not appear in the Acknowledgments section or other areas of your manuscript. We will only publish funding information present in the Funding Statement section of the online submission form.

Please remove any funding-related text from the manuscript and let us know how you would like to update your Funding Statement. Currently, your Funding Statement reads as follows: "This study was partially financed by the Fundação de Apoio à Pesquisa e a Inovação Tecnológica do Estado de Sergipe (Fapitec/SE/Brasil) [Research and Technological Innovation Support Foundation in the State of Sergipe] and the Coordenação de Aperfeiçoamento de Pessoal de Nível Superior - Brasil (CAPES/Brasil) [Coordination for the Improvement of Higher Education Personnel], financing code 001, PROMOB, process no. 88881.157882/2017-01.

<https://www.gov.br/capes/pt-br>
<https://fapitec.se.gov.br/>

The funders had no role in study design, data collection and analysis, decision to publish, or preparation of the manuscript."

Please include your amended statements within your cover letter; we will change the online submission form on your behalf.

**Response:** The suggested changes have been made.

# Acknowledgements

We thank all the participants who agreed to participate in this study. We also thank TMSV, GBC, RKFS, ACOB, Hypertension Nutrition Care Group (*Grupo de Atendimento Nutricional em Hipertensão*, GANuH), and Nutrition Laboratory (*Laboratório de Nutrição*, LABNUT) for assistance in collecting data and analyzing biological samples.

8. In your Data Availability statement, you have not specified where the minimal data set underlying the results described in your manuscript can be found. PLOS defines a study's minimal data set as the underlying data used to reach the conclusions drawn in the manuscript and any additional data required to replicate the reported study findings in their entirety. All PLOS journals require that the minimal data set be made fully available. For more information about our data policy, please see <http://journals.plos.org/plosone/s/data-availability>.

Upon re-submitting your revised manuscript, please upload your study’s minimal underlying data set as either Supporting Information files or to a stable, public repository and include the relevant URLs, DOIs, or accession numbers within your revised cover letter. For a list of acceptable repositories, please see <http://journals.plos.org/plosone/s/data-availability#loc-recommended-repositories>. Any potentially identifying patient information must be fully anonymized.

Important: If there are ethical or legal restrictions to sharing your data publicly, please explain these restrictions in detail. Please see our guidelines for more information on what we consider unacceptable restrictions to publicly sharing data: http://journals.plos.org/plosone/s/data-availability#loc-unacceptable-data-access-restrictions. Note that it is not acceptable for the authors to be the sole named individuals responsible for ensuring data access.

We will update your Data Availability statement to reflect the information you provide in your cover letter.

**Response:** The database containing the collected data will be attached.

9. We note that you have stated that you will provide repository information for your data at acceptance. Should your manuscript be accepted for publication, we will hold it until you provide the relevant accession numbers or DOIs necessary to access your data. If you wish to make changes to your Data Availability statement, please describe these changes in your cover letter and we will update your Data Availability statement to reflect the information you provide.

**Response:** The suggested changes have been made.

10. Please ensure that you include a title page within your main document. You should list all authors and all affiliations as per our author instructions and clearly indicate the corresponding author.

**Response:** The suggested changes have been made.

11. Please amend either the abstract on the online submission form (via Edit Submission) or the abstract in the manuscript so that they are identical.

**Response:** The suggested changes have been made.

12.Please include captions for your Supporting Information files at the end of your manuscript, and update any in-text citations to match accordingly. Please see our Supporting Information guidelines for more information: <http://journals.plos.org/plosone/s/supporting-information>.

**Response:** The suggested changes have been made.

Reviewers' comments:

Reviewer #1: The article addresses an important field of nutritional research. Evaluating the relative validity of a dietary assessment method is of great importance when studying the potential impact of diet exposures on health outcomes. The authors constructed an FFQ for foods high in sodium and proposed a validation protocol over a 6-month period of dietary data collection complemented by four 24-hour recalls and two 24-hour urinary excretion for sodium analysis. The FFQ was reproductible but not valid. Despite the good statistical procedures adopted, the writing is not always fluid and sometimes repetitive. The description of the study protocol is not clear. In addition, it is not always clear what each test adds to the overall evaluation of level of validity, which could be made clearer in the method section. Another concern I have is related to the 'per se' construction of FFQ that was done previously with another sample; so, in the middle of the methodology, the authors describe basically another study (not mentioned before) and confuse the reader. Important decisions on how FFQ was built are not provided in the text.

Very important: The English needs considerable attention.

**Response:** For validation, the triad method was used by triangulation between the FFQ-FHS, 24hR, and biomarker. For reproducibility, the intraclass coefficient of correlation (ICC) and Kappa coefficient were used; Bland–Altman plots were constructed to graphically demonstrate the level of agreement between the FFQ-FHS, 24hR, and biomarker.

More information about the constructed FFQ-FHS has been added to the methodology.

Introduction:

1. I suggest deleting the first two paragraphs and getting straight to the point. In the last paragraph, authors could justify why this study was done and, especially to investigate sodium in that region of Brazil. (why a specific FFQ is needed to that region)

**Response:** The suggested changes have been made. The two paragraphs have been deleted, and the justification has been inserted in the last paragraph.

In Brazil, independent of the geographical region, the sodium intake of the population is high [11,12] and corresponds to approximately twice the intake recommended by the World Health Organization (WHO) (2 g/day) [13]. Data from the 2008 to 2009 Household Budget Survey show that the contribution of processed and ultra-processed foods to the average sodium intake increased from that observed in the first edition of the survey (2002–2003) [11]. Despite this, studies seeking the validation of a tool to investigate sodium intake in the Brazilian population, especially in the northeast region, remain scarce.

Methods

2. I believe that the authors could benefit from checking the manuscript construction of previous FFQ validation studies. As mentioned above, the writing is not fluid and sometimes repetitive. First, provide an overall description of the (i) previous protocol to design the FFQ; (ii) the 6-month protocol to the FFQ validation: FFQ, rec24h, urine collection (in a 6-month period, when each instrument was applied/data was collected, providing the interval period among them).

**Response:** The suggested changes have been made.

3. In the sentence: “Each volunteer answered three 24hR, with a minimum and maximum interval of 15 and 45 days, respectively, and one of 24hR covered one weekend day.” How was the 15-45-day interval decided? Does it depend on the availability of the participant?

**Response:** Each volunteer answered three 24hR questions, with a minimum and maximum interval of 15 and 45 days, respectively, considering the participants' availability, including one day during the weekend.

4. Do FFQ and Rec24h were paper-based methods? Were both a face-to-face interview?

**Response:** Yes, all interviews regarding the application of the R24h and FFQ instruments were conducted face-to-face.

5. Very important: do not describe “researchers” when referring to those people that collected the data in the field work; in this case, I would recommend to describe they as “research assistants” or “assistants”. It is better to restrict the term 'researchers' to those responsible for the study protocol and who wrote the manuscript.

**Response:** The suggested changes have been made.

6. How many research assistants and nutritionists conducted the field work? Was the team composed of undergrad and grad students? Those who applied the dietary instruments had any background in nutrition & dietetics?

**Response:** All four assistants were nutrition graduate students who received training and supervision from the nutritionists.

7. Examples of information that seems repetitive in the text:

(a) All the subjects who agreed to participate in the study received information on the study and signed the Free and Informed Consent Form.

(b) The participants signed the Free and Informed Consent Form, allowing their voluntary participation,

(a) All researchers had been trained and supervised by nutritionists.

(b) Nutritionists trained and supervised the anthropometric measurements to ensure higher precision and accuracy of the data.

**Response:** The suggested changes have been made.

8. Rewrite the sentence: To assess daily sodium intake, to the 24hR estimate was added the amount of sodium from added salt.

**Response:** The suggested changes have been made.

9. Briefly describe the data collected from the socioeconomic questionnaire applied. Age, sex, occupation, etc.

**Response:** A structured questionnaire was used to collect sociodemographic information (sex, age, household per capita income, and educational level).

10. Provide more details on the multiple pass method applied in the current protocol.

**Response:** The MPM is an approach technique for applying 24hR, aimed at increasing the accuracy of information. The MPM was proposed in five steps.

1 Quick list
2 Forgotten foods list

3 Time and occasion

4 Detail and review

5 Final review

11. Validation paragraph: It does not add additional information. In the way described, this paragraph can be deleted.

**Response:** The suggested changes have been made.

12. How was the information of the dietary intake of rec24h and FFQ entered in NutWin? Food intake data entered one-by-one? who and how many people entered these data? There was any quality control in the data entered? how portion sizes were defined? The same questions is applied to FFQ.

**Response:** To facilitate the completion of all forms, a photographic manual of food quantification with a preset portion size was used.

The standard portion size of each food or meal was defined according to the Brazilian Institute of Geography and Statistics (*Instituto Brasileiro de Geografia e Estatística*, IBGE) database of reference measurements and portion sizes of foods and dishes consumed in Brazil.

The food nutrient composition was analyzed using NutWin® software (version 1.5.2.51). All items listed in the applied 24hR and FFQ-FHS were standardized and typed individually in duplicate.

13. Describe more about the MSM. What this method proposes and how?

**Response:** More information about the MSM has been included in the statistical analysis section.

Results

14. It calls my attention that the sample studied had higher education but low income. Who were they? Undergrad students or staff personnel? It is important to describe it in method section.

**Response:** The suggested changes have been made.

15. Table 1. Replace 'gender' by 'sex'. sex = biologic nature of being female or male. gender = social role construction of being male or female. If you are analyzing urinary excretion, it is more relevant that you provide the information on biology. Also, replace 'nutrition status' by 'body mass index (kg / m2)'.

**Response:** The suggested changes have been made.

16. Replace “CI95%” by 95% CI

**Response:** The suggested changes have been made.

17. I think authors can think about a better presentation on tables.

**Response:** The suggested changes have been made.
