# Supplementary material for: Food frequency questionnaire for foods high in sodium: Validation with the triads method
Source: PLoS One. 2023 Jul 3;18(7):e0288123. doi: 10.1371/journal.pone.0288123 (PMC10317220; doi:10.1371/journal.pone.0288123)
Supplement: Attachment — Submitted filename: 2. Response_to_Reviewers.docx [file pone.0288123.s003.docx]

Response to Reviewers

Methods:

Sodium biomarkers:

1. Please describe the instructions provided for participants of how to collect 24-h urine samples. Also, provide a reference for it.

**Response:**

A 24-hour urine collection was adopted as an instrument to assess creatinine clearance and estimate Na+ electrolyte intake. Participants were instructed regarding urine collection and received a 2 L plastic bottle. Furthermore, the participants received a questionnaire containing written information about the correct urine collection procedure to be completed and delivered on the scheduled day. The following collection instructions were standardized:

- At the beginning of the collection, the participant discarded all urine stored in the bladder in the toilet. From then on, the participant stored all urine, including night and bath urine, in the bottle until the next day.

- The collected urine wasstored in a refrigerator, both during and after collection.

Reference: Sociedade Brasileira de Patologia Clínica/Medicina Laboratorial(SBPC/ML). Recomendações da Sociedade Brasileira de Patologia Clínica/Medicina Laboratorial (SBPC/ML): coleta e preparo da amostra biológica. Barueri, SP: Manole: Minha Editora. 2014: 487p.

2. The reference methods applied to verify the completeness of urine samples are not clear. First, is there a reference of less than 400 ml/day for completeness? Second, the study of Charlton KE et al 2008 (21) used multiple parameters, as volume of urine less than 500 ml/day and creatinine 0.2–0.3 mmol kg/day. These parameters and references are very important considering that urine provided the biomarker. If author be more rigid to accept a sample as valid (e.g., equal or more 500 ml/day + creatine output >0.2 mmol kg/day), how many samples would be valid in both methods? Did you test whether concordance with dietary method had improved?

**Response:**

For the metabolites to be excreted, a minimum urine volume of 400mL is required, according to Nemeret al. (2010). For this research, urine samples lessthan 400mL and/or those whose collection period exceeded the determined 24 h were excluded. Creatinine excretion was also analyzed as an indicator of adequate urine collection. Urine samples with creatinine levels<0.2 mmol/kg/day were excluded. The results were converted to mg/kg/day (CHARLTON, et al., 2008).

When performing urinary creatinine analysis, only one participant met the exclusion criterion of creatinine <0.2 mmol/kg/day, obtaining a result of 0.171 mmol/kg/day in the second urine sample collected.

3. Sociodemographic data. Please indicate the currency conversion of Brazilian currency to USD$ at the time of the study.

**Response:**

The suggested changes have been made.

4. Anthropometric measurements: indicate the equipment (registered brand) of each anthropometric tool (electronic scale, stadiometer, and tape)

**Response:**

Digital platform scale, with 100g precision (LIDER®, São Paulo, Brazil)

Portable Stadiometer, with 0.1 cm resolution (Alturaexata®, Minas Gerais, Brazil)

5. Food composition analysis of FFQ: Has NutWin a specific interface for FFQ calculation? Or were all food items converted to daily portion size in order to be entered in this software? Please, describe in methods.

**Response:**

For the analysis of the nutritional composition of the food frequency questionnaire (FFQ), all items were converted into individual consumption portions and subsequently tabulated using the software.

6. Statistics: Which method was applied to calculate energy adjustment?

**Response:**

Sodium intake was adjusted using the residual method to control for confounding factors and remove external oscillations, according to Willetet al. (1997). Simple linear regression analysis was performed, considering energy as an independent variable and sodium as a dependent variable.

7. Line 200: Correct typing mistake “With Rregard to nutritional 200 status…”

**Response:**

The suggested changes have been made.

8. Table 1: present variables summarized as mean first, then after the categorical ones; Use only 1 decimal to summarize age

**Response:**

The suggested changes have been made.

9. Results: Line 209-211: Adequacy in urine sample collection: First, there is no need to repeat the parameter in parenthesis already described in methods; second, indicate how many were adequate according to volume and how many according to creatinine value. Also, as described above, test whether being more rigid in urine assessments could improve the final results with dietary methods.

**Response:**

Considering a urinary volume of ≥ 500mL, 43 and 36 urine samples from the first collection and second collections, respectivelywere considered adequate. By joining two exclusion criteria (e.g., equal or more 500 mL/day + creatine output >0.2 mmol kg/day), 43 urine samples from the first collection and 32 samples from the second collection were considered adequate.

New statistical tests were performed on the 32 urine samples. The table below presents the results.

**Table 1.** Spearman’s correlation and validity coefficients for unadjusted and energy-adjusted daily sodium intake in adults in the academic community. Aracaju, SE, 2018 (n=50)

| **Correlation coefficient** | | | **Validity coefficient** | |
| --- | --- | --- | --- | --- |
|  | **Unadjusted (p)** | **Adjusted (*p*)*** |  | **Adjusted*** |
| ***r*FFQR** | 0.386 (<0.01) | 0.129 (0.387) | **ρFFQAI** | 0.26 |
| ***r*RB**† | −0.026 (0.882) | 0.172 (0.316) | **ρRAI**† | 0.85 |
| ***r*FFQB**† | 0.014 (0.936) | 0.052 (0.763) | **ρBAI**† | 0.20 |

*r*FFQR, correlation between the food frequency questionnaire survey and the 24-h dietary recall

*r*RB, correlation between the 24-h dietary recall and biomarker assessment

*r*FFQB, correlation between the food frequency questionnaire survey and biomarker assessment

ρFFQAI, validity coefficient of the food frequency questionnaire survey

ρRAI, validity coefficient of the 24-h dietary recall

ρBAI, biomarker validity coefficient

$$\rho FFQAI=\surd([rFFQR x rFFQB])/r\mathrm{RB}$$

$$\rho RAI=\surd([rFFQR x rRB])/r\mathrm{FFQB}$$

$$\rho BAI=\surd([rRB x rFFQB])/r\mathrm{FFQR}$$

*Daily energy-adjusted sodium intake

†n,36 using the average urinary sodium excretion

**Table 2.** Spearman’s correlation and validity coefficients for unadjusted and energy-adjusted daily sodium intake in adults in the academic community. Aracaju, SE, 2018 (n=32)

| **Correlation coefficient** | | | **Validity coefficient** | |
| --- | --- | --- | --- | --- |
|  | **Unadjusted (p)** | **Adjusted (*p*)*** |  | **Adjusted*** |
| ***r*FFQR** | 0.242 (0.182) | 0.244 (0.178) | **ρFFQAI** | 0.23 |
| ***r*RB**† | 0.139 (0.449) | 0.232 (0.201) | **ρRAI** | 1.06 |
| ***r*FFQB**† | 0.225 (0.215) | 0.050 (0.786) | **ρBAI** | 0.22 |

The repetition of statistical tests demonstrates that even after considering new inclusion parameters for adequate urine samples (e.g., equal or more 500 ml/day + creatine output >0.2 mmol kg/day), the correlations and validity coefficients remained insufficient for the FFQAI validation.
