# Supplementary material for: Food frequency questionnaire for foods high in sodium: Validation with the triads method
Source: PLoS One. 2023 Jul 3;18(7):e0288123. doi: 10.1371/journal.pone.0288123 (PMC10317220; doi:10.1371/journal.pone.0288123)
Supplement: Attachment — Submitted filename: Response to Reviewers.doc [file pone.0288123.s004.doc]

**Response to Reviewers**

I just request some minor corrections in the text, and 1-2 other adjustments in tables and text.

Please add spacing in the following passages:

Line 42: “However, Bshave limitations…” = B have
Line 43: “…Blevels…” = B levels
Line 47: “… 24hR or FR, and Bs,…” = B (all other instruments described in this sentence are in singular version)
Line 58: “… to2009…”
Line 62: “… remainscarce”
Line 75: “… 2018.Fifty…”
Line 85: “… period of 6months..” = 6 months
Line 89: Fig1 Data collectionprocess
Line 94: “…24hR and FFQ-FHS survey,” = there is no need the term “survey”
Line 96: “… was completed after the 24hRviaface-to-face…”
Line 123: “After 3months…” = 3 months
Line 124: “…24hRsusing…”
Line 140: “[10]to determine”
Line 164: “…precision of 1millimeter” = 1 millimeter
Line 325: “… notwithstanding…”
Line 370: “… duringin-person interviews,…”
Line 386: “… over a period of 6months” = 6 months
Line 77: Please, reedit the sentence: “We excluded pregnant and lactating women…"

**Response:**

The requested corrections have been made.

Other minor corrections:

Please review in all Tables and Bland and Altman figure the following: Clear state in the tables when the “adjusted” means “energy adjusted”, otherwise it means traditional statistical adjustments for covariates.

**Response:**

The suggested changes have been made.

Line 296: “… which eventually lost significance after adjustment.” Does it mean energy adjustment? Please specify in the text.

**Response:**

Yes, does it mean energy adjusment. The suggested changes have been made.
